# Dataset on the characteristics of the liquid effluent issued from separation of faeces and urine under slats using V-shaped scraper in swine buildings

**DOI:** 10.1016/j.dib.2020.105533

**Published:** 2020-04-11

**Authors:** Clément Likiliki, Bertrand Convers, Fabrice Béline

**Affiliations:** a Cooperl Environnement, Lamballe, France; b INRAE, UR OPAALE, Rennes Cedex, France

**Keywords:** Physicochemical characteristics, V-shaped scraper, Liquid effluent, Urine

## Abstract

Separation of faeces and urine under slats in swine buildings using different technologies such as V-shaped scraper is becoming popular. Indeed, such a separation can allow improving air quality in the building and can also facilitate management of manure through concentration of carbon and phosphorus in the solid phase. Though solid phase management is well established through anaerobic digestion, the liquid phase, mainly urine, is still to be recycled or disposed of. However, the characteristics of this kind of effluent are quite different compared to slurry obtained from conventional building and corresponding to a mix of urine and faeces. Such characteristics are poorly documented and very few data are available in the literature. This dataset provides insight on the characteristics of this effluent to achieve treatment or nutrient recovery. Nine pig farms equipped with a V-shaped scraper and using different farming practices were selected for the sampling of this liquid phase. Building characteristics including animal capacity and physiological stage of animals and feeding strategy were taken into account. The characterisation of this effluent focused on global parameters (pH, Alkalinity, TS, VS, TSS, VSS) but also on the carbon and nitrogen content (COD, TKN, TAN). Total compounds and dissolved ions were also analysed to round off the characterisation. Finally, the characteristics of such liquid effluent were compared to urine collected directly from animals without contact with faeces.

Specifications table

**Table:** 

Subject	Environmental chemistry
Specific subject area	Waste characterisation
Type of data	Tables Figures
How data were acquired	Data were acquired using classical physico-chemical analyses and instruments including: pH probe, oven drying, furnace calcination, mineralisation, titration, ionic chromatography, photo spectrometry and Microwave Plasma-Atomic Emission Spectrometer.
Data format	Raw data and statistical treatment
Parameters for data collection	Characterisation data were obtained from nine liquid samples collected in 2019 from nine pig farms equipped with a V-shaped scraper. The liquid phase was collected either at the exit of the barn or in the storage tank.
Description of data collection	After collection, samples were stored at 4 °C until analyses. Analyses were performed within 3–5 days after collection. Centrifugation (13,700G for 20 min at ambient temperature) and filtration on 0.45 µm PP+GF syringe filters was performed before determination of soluble characteristics.
Data source location	Samples were collected in pig farms around the city of Rennes (48°06′053″N, 1°40′046″W), in both the counties of Ille-et-Vilaine and Côtes-d'Armor (Bretagne, France)
Data accessibility	With the article

## Value of the data

• The dataset provides the average physico-chemical characteristics of the liquid effluent coming from the separation of faeces and urine under slats using V-shaped scraper in swine buildings. This dataset also shows the variations of these characteristics from one site to another. Such technology allowing solid/liquid separation in the building is quite recent and, consequently, characteristics of the products are poorly documented.
• These data could prove useful for researchers and engineers to design and study strategies for nutrient recovery or disposal from the liquid phase issued from separation of faeces and urine under slats using V-shaped scraper in swine buildings.
• Moreover, these data can benefit researchers and engineers to consider the agronomic value of such effluent. Eventually, farmers equipped with this technology can get benefits from these data for improvement of its management.
• In future, those data could be a part of a database on characteristics of the liquid effluent issued from separation of faeces and urine which could prove useful for agronomic, engineering or modelling purposes.

## 1 Data description

In-barn separation of faeces and urine displays many advantages. Firstly, the continuous removal of the liquid phase reduces ammonia and nitrite oxide emissions, up to 54% and 49% respectively, improving the indoor air quality [1]. Secondly, it allows concentrating 55% of total nitrogen and 91% of phosphorus content in the solid phase [1]. Finally, the valorisation of the solid fraction through anaerobic digestion enables the production of methane and thus reduces greenhouse gases emissions but also nitrogen losses [2]. Although, the management of the solid phase is rooted, the liquid phase is still to be handled. A better knowledge of its characteristics could help to establish or optimize recovery or treatment processes.

In order to have a representative selection of pig buildings equipped with a V-shaped scraper in terms of building characteristics and farming practices, nine pig farms were chosen for the implementation of the sampling (Table 1). Depending on the building setup, samples were either collected raw or from the storage tank.

**Table 1:** Pig farms capacities (places on top of the scraper), feeding strategy and sampling point.

Pig farm no.	Building capacity (animal number)		Feeding strategy		Sampling point
	Fattening	Post weaning	Feed production	Feeding system	
1	900	0	On-farm	Liquid	Raw
2	672	0	On-farm	Liquid	Raw
3	750	0	On-farm	Liquid	Raw
4	360	0	On-farm	Liquid	Raw
5	750	750	Purchased complete	Dry	Raw
6	780	0	Purchased complete	Liquid	Tank bottom
7	1080	0	On-farm	Liquid	Tank supernatant liquid
8	1400	0	On-farm	Liquid	Tank supernatant liquid
9	720	400	Purchased complete	Liquid (fattening)/ Dry (post weaning)	Tank supernatant liquid

The physicochemical characteristics of the liquid phase issued from this separation are displayed in Table 2, Table 3, Table 4, Table 5, providing average data and also data from each farm.

**Table 2:** pH, Alkalinity (gCaCO3/L), Total Solids, Volatile Solids, Total Suspended Solids and Volatile Suspended Solids (g/L).

Pig Farm no.	pH	Alkalinity	TS	VS	TSS	VSS
1	9.08	4.583	13.79	8.73	8.17	6.38
2	9.14	9.250	23.22	12.20	7.62	5.50
3	7.71	2.833	13.11	7.71	3.13	2.66
4	9.11	10.083	18.46	7.47	3.54	2.76
5	8.91	8.000	17.87	9.79	7.68	5.13
6	7.97	4.500	13.90	8.33	7.71	5.16
7	7.98	11.417	9.76	3.64	1.07	0.75
8	7.66	11.917	11.42	4.56	2.26	1.35
9	7.92	7.000	8.38	3.18	1.08	0.59
Mean	8.39	7.731	14.43	7.29	4.70	3.36
Standard deviation	0.65	3.238	4.68	2.99	3.05	2.22

**Table 3:** Total Chemical Oxygen demand and Soluble Chemical Oxygen Demand (gCOD/L) and Total Kjeldahl Nitrogen and Total Ammonia Nitrogen (gN/L).

Pig Farm no.	Total COD	Soluble COD	TKN	TAN
1	14.02	8.66	2.34	1.42
2	24.78	17.33	4.15	3.06
3	13.68	10.45	1.43	0.54
4	14.40	9.99	3.16	2.13
5	14.99	14.87	4.13	2.52
6	4.07	2.20	1.48	0.53
7	8.42	8.00	3.39	2.66
8	9.69	9.13	3.64	2.78
9	4.75	4.28	1.93	1.24
Mean	12.09	9.43	2.85	1.88
Standard deviation	6.31	4.68	1.08	0.97

**Table 4:** Total compounds content (mg/L).

Pig Farm no.	Ca	Mg	Cu	Zn	P
1	191.8	154.0	1.3	4.9	218.8
2	216.5	269.1	1.6	6.6	276.2
3	212.6	163.2	0.9	3.5	117.0
4	126.6	55.7	1.0	4.0	121.9
5	213.5	219.4	1.8	4.8	198.9
6	443.2	161.2	7.2	11.8	213.4
7	116.7	34.5	0.6	1.8	75.4
8	137.5	27.6	0.7	2.0	56.4
9	87.7	25.0	0.8	1.8	41.1
Mean	194.0	123.3	1.8	4.6	146.5
Standard deviation	105.1	90.7	2.1	3.1	83.0

**Table 5:** Dissolved ions content (mg/L).

Pig Farm no.	Cl	P-PO4	S-SO4	Na	K	Ca	Mg
1	837.6	2.1	258.2	368.1	1331.5	113.7	16.5
2	2494.2	0.0	611.9	1430.6	2866.9	107.7	55.5
3	877.4	6.5	187.6	589.3	1182.1	120.8	84.6
4	1915.1	34.7	296.7	1126.3	3045.2	44.2	12.3
5	1862.4	0.0	408.2	471.7	2203.8	53.4	76.7
6	620.7	12.1	1.0	245.9	930.1	102.5	34.0
7	1182.6	24.2	67.0	616.5	1797.3	81.1	24.9
8	1384.2	30.9	54.7	761.4	1980.9	101.0	26.6
9	1198.7	25.7	1.3	501.4	1498.2	95.9	26.2
Mean	1374.7	15.1	209.6	679.0	1870.7	91.1	39.7
Standard deviation	607.8	13.9	207.1	377.9	732.2	26.5	26.3

Additionally, five samples were collected throughout the year 2019 from the pig farm no. 1 to assess for seasonal variations. Physicochemical characteristics over that year are presented in Table 6.

**Table 6:** Variations of effluent characteristics over the year 2019 (farm no. 1).

	16/01 (1_1)	31/01 (1_2)	01/04 (1_3)	02/07 (1_4)	04/11 (1_5)	Mean	Standard deviation
pH	8.88	9.01	8.66	8.68	8.62	8.77	0.17
Alkalinity	10.394	8.323	11.573	7.359	5.287	8.587	2.482
TS	19.08	18.47	24.16	13.12	16.43	18.25	4.04
VS	12.03	11.36	14.80	6.79	8.96	10.79	3.05
TSS	9.32	10.96	11.28	4.84	5.49	8.38	3.04
VSS	7.36	8.21	8.40	3.49	3.95	6.28	2.38
Total COD	20.79	17.74	28.81	17.30	18.94	20.72	4.72
Soluble COD	14.66	14.71	17.26	10.81	12.53	13.99	2.44
TKN	4.32	4.71	4.91	2.79	3.44	4.03	0.90
TAN	2.54	3.95	3.09	2.08	2.22	2.78	0.76
Cl	1243.3	887.0	1213.4	1253.1	1321.5	1183.7	170.5
P-PO4	0.5	14.7	6.6	1.9	0.6	4.8	6.0
SO4	607.5	585.5	611.1	444.5	719.0	593.5	98.1
Na	541.2	438.2	665.4	690.9	782.4	623.6	134.8
K	1870.9	1593.6	2414.2	2080.1	2767.7	2145.3	459.2
Ca	71.9	44.8	130.7	85.0	95.1	85.5	31.5
Mg	17.8	11.4	23.5	47.0	100.1	39.9	36.2

Finally, to assess the difference between this liquid effluent and pig urine, a sample was directly collected from pigs in metabolic cages. Therefore, faeces and urine were never in contact unlike the liquid phase issued from a pig house equipped with a separation system. The main characteristics of this urine are displayed in Table 7. To facilitate the comparison between both effluents, the mean value of the liquid phases collected raw is also mentioned.

**Table 7:** Main characteristics of pig urine.

	Pig urine	Mean value of the liquid phases sampled raw		Pig urine	Mean value of the liquid phases sampled raw
pH	9.30	8.79	Cl	1794.4	1597.3
Alkalinity	13.276	6.950	P-PO4	837.5	8.7
			S-SO4	89.5	352.5
Total COD	4.93	16.37	Na	1820.0	797.2
Soluble COD	4.39	12.26	K	1812.3	2125.9
TKN	3.80	3.04	Ca	82.0	88.0
TAN	3.77	1.93	Mg	23.0	49.1

**Fig. 1:**
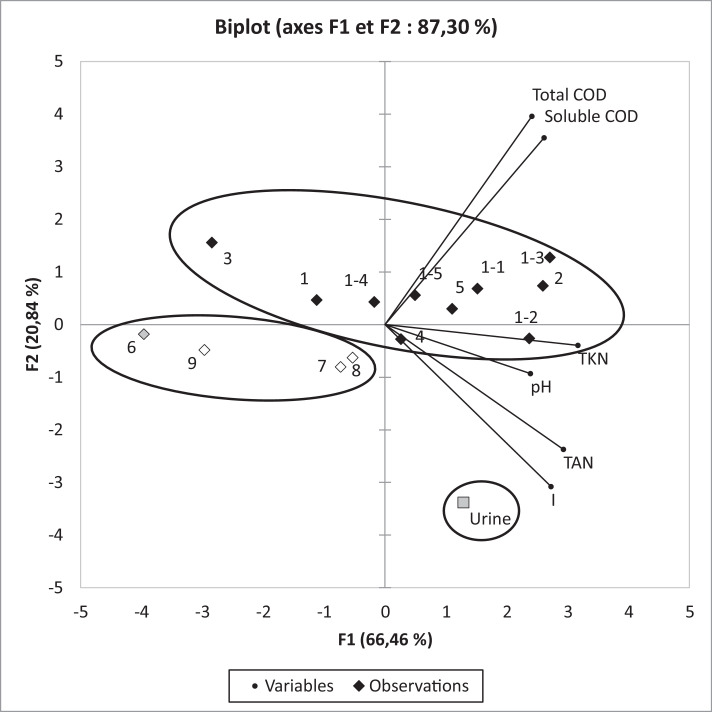
Biplot from PCA analysis of all the samples collected (grey square: urine sample, grey diamond: bottom of tank sample, empty diamonds: tank supernatant samples; I: ionic strength).

To determine the correlations between farming practices, sampling points and the physicochemical characteristics of the liquid effluent, a Principal Component analysis (PCA) was performed. Only parameters available for all samples including urine were chosen for this PCA analysis. The ionic strength calculated from dissolved ions content was used as parameter rather than all dissolved ions. Finally, only F1 and F2 eigenvectors with an eigenvalues greater than one were considered for plotting the PCA biplot graph (Fig. 1). Both F1 and F2 components explain 87.3% of variance.

This statistical analysis corroborates the difference between pig urine and the liquid effluents from pig farms equipped with V-shaped scrapers. Additionally, sampling points also differentiate effluents through this statistical analysis whereas farming practices are not identified as an influencing parameter.

## 2 Experimental design, materials, and methods

### 2.1 Sampling

Samples from the different pig farms were collected on a one-off basis between January and February 2019. Pig farm n° 1 was chosen to assess for the variations over a year. These variations were taken into account through 5 sampling spread over the year 2019, from January to November.

The liquid phases were collected either from the pipe going from the building to the storage tank (raw) or directly inside the storage tank when the pipe was not accessible. The samples collected raw consisted in gathering 2 L of liquid phase coming out of the building at the time of the sampling. When taken from the storage tank, it should be noted that no mixing system was set up in the tank. Therefore, samples were either supernatant liquid or settled sludge at the bottom as indicated in Table 1. All samples were collected in the morning.

The sample of pig urine was collected from four pigs in metabolism cages fed with purchased complete feed in liquid form. Thanks to the display of those cages, urine and faeces can be collected from different spots and therefore never be in contact. The sample was a mix of the urine produced during one day from the four pigs.

Samples were stored at 4 °C until analyses.

### 2.2 Analyses

#### 2.2.1 Physicochemical analyses

Analyses were carried out within 3–5 days after collection. Centrifugation (13,700G for 20 min at ambient temperature) and filtration on 0.45 µm PP+GF syringe filters were performed before determination of soluble characteristics. pH was measured using a pH-meter (pH197i, WTW) and alkalinity using the titration method (Titralab AT1000 Series, Hach Lange). TS, VS, TSS and VSS were calculated according to standard methods (NF EN 12880, NF EN 15935 and NF T90-105-2 respectively).

COD was determined by colorimetric method using LCK014 cuvette tests from Hach Lange. Mineralisation was performed with a LT2000 and absorbance reading with a DR3900 spectrophotometer (Hach Lange). Standard method NF EN 13342 was used for TKN measurements (Kjeldahltherm 20 s for mineralisation and Vapodest 50 s for distillation, Gerhardt).

Total Ca, Mg, Cu and Zn were assessed through Microwave Plasma-Atomic Emission Spectrometer (MP-AES 4200 Agilent technologies) after mineralisation of the ashes produced from the VS determination. For total phosphorus the analysis was achieved by a discrete photometric analyser (Gallery, Thermo Scientific) also after mineralisation.

Dissolved ions and TAN were analysed with the 850 Professional Ion Chromatography from Metrohm. Nitrites and nitrates are not presented since none were found in the samples.

#### 2.2.2 Statistical analysis

A Principal Component Analysis (PCA) was performed thanks to the XLStat 2018 software as an add-in to Excel. 15 observations were used; when taking into account the 9 samples from the different pig farms, the 5 samples collected throughout a year from the farm no. 1 and the pig urine. In order to keep the number of variables lower than the observations, dissolved ions and alkalinity were combined as one variable: the ionic strength. Variables used for these statistical analyses were pH, total and soluble COD, total and ammoniacal nitrogen content and ionic strength.

## Conflict of interest

The authors declare that they have no known competing financial interests or personal relationships which have, or could be perceived to have, influenced the work reported in this article.
